# Tirabrutinib-associated toxic epidermal necrolysis: a case report and review of the literature

**DOI:** 10.1093/skinhd/vzaf011

**Published:** 2025-04-04

**Authors:** Noriko Ikegawa, Natsuko Saito-Sasaki, Yu Sawada

**Affiliations:** Department of Dermatology, University of Occupational and Environmental Health, Kitakyushu, Japan; Department of Dermatology, University of Occupational and Environmental Health, Kitakyushu, Japan; Department of Dermatology, University of Occupational and Environmental Health, Kitakyushu, Japan

## Abstract

Tirabrutinib, a Bruton tyrosine kinase (BTK) inhibitor, has shown efficacy in recurrent or refractory diffuse large B-cell lymphoma (DLBCL). However, severe drug eruptions, including toxic epidermal necrolysis (TEN), have been reported. We present a case of a 74-year-old man with refractory DLBCL who developed TEN after 1 month of tirabrutinib treatment. Severe drug eruptions linked to tirabrutinib are rare, with only three cases reported, including ours. Tirabrutinib’s selective BTK inhibition may activate cytotoxic CD8^+^ T cells, contributing to Stevens–Johnson syndrome/TEN pathogenesis. Further studies are needed to clarify the mechanisms and develop better diagnostic approaches for BTK inhibitor-related drug eruptions.

## Teaser

Tirabrutinib, a BTK inhibitor, is effective for refractory DLBCL but may cause severe eruptions like TEN. We report a case of TEN treated successfully with steroids and IVIg despite negative LST. The findings highlight a need for targeted strategies to identify and manage tirabrutinib-induced TEN.

**What is already known about this topic?**

- Tirabrutinib, a selective Bruton tyrosine kinase (BTK) inhibitor, is effective in treating recurrent or refractory diffuse large B-cell lymphoma.
- Severe drug eruptions, including Stevens–Johnson syndrome and toxic epidermal necrolysis (TEN), are rare but potentially life-threatening adverse effects.

**What does this study add?**

- This study presents a rare case of tirabrutinib-induced TEN, providing clinical and histopathological insights into its presentation and management.
- It also suggests a potential role of BTK inhibition in activating cytotoxic CD8^+^ T cells, contributing to the pathogenesis of TEN.

Tirabrutinib, a Bruton tyrosine kinase (BTK) inhibitor, has been used for recurrent or refractory diffuse large B-cell lymphoma (DLBCL).^1^ It has demonstrated a high overall response rate in recurrent or refractory DLBCL, with response rates ranging from 70% to 77% and complete response rates between 23% and 38%. However, in domestic phase I/II trials, grade 3 or higher erythema multiforme was observed in 6.8% of patients treated with tirabrutinib, raising concerns about severe drug eruptions.^2^ Here, we report a case of toxic epidermal necrolysis (TEN) caused by tirabrutinib and present a review of previously reported cases.

## Case report

**Figure 1:**
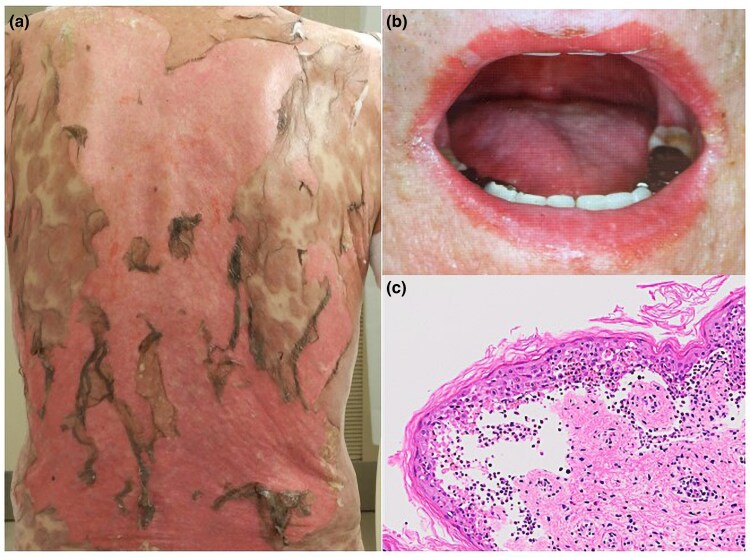
Clinical manifestation and histological examination. (a, b) Clinical manifestations. (a) Extensive erosions covering 30% of the body surface area, primarily on the trunk, along with (b) mucosal lesions in the lip and oral cavity. (c) Histological examination. Skin biopsy showing keratinocyte necrosis in the epidermis, subepidermal blistering and eosinophil infiltration in the dermis (× 10 magnification).

A 74-year-old man was undergoing treatment for DLBCL at the Department of Hematology, Hospital of the University of Occupational and Environmental Health, Kitakyushu, Japan. Due to the refractory nature of the disease, tirabrutinib (480 mg daily) was initiated. One month after starting treatment, the patient developed pruritic light-red patches on his trunk and thighs, along with blisters on his lips. He was referred to our dermatology department for evaluation of the rash. The patient had no constitutional symptoms, such as fever, malaise or myalgia, before the onset of skin and mucous membrane involvement. On examination, he had extensive erosions covering 30% of his body surface area, primarily on the trunk, and mucosal lesions in the oral cavity and lip (Figure 1a, b). The patient’s body temperature at admission was 39.8 °C. Laboratory tests showed white blood cell count 3.4 × 109 cells L^–1^ without atypical lymphocytes, aspartate aminotransferase 20 U L^–1^, alanine aminotransferase 14 U L^–1^ and C-reactive protein 0.85 mg dL^–1^. Tests for anti-BP180 antibodies, herpes virus, Epstein–Barr virus and cytomegalovirus were negative. A skin biopsy revealed keratinocyte necrosis in the epidermis, subepidermal blistering and eosinophil infiltration in the dermis (Figure 1c). Based on these clinical and histopathological findings, the patient was diagnosed with TEN.

The tirabrutinib was discontinued, and the patient was treated with steroid pulse therapy for 3 days, followed by prednisolone at 1000 mg daily for 3 days, along with high-dose intravenous immunoglobulin (IVIg) (400  mg kg^–1^ daily) for 5 days, with each IVIg infusion administered over 3 h. SCORTEN at presentation was 3, and on the third day, it remained 3, although some parameters were not measured. At presentation, the clinical and laboratory parameters used for SCORTEN were as follows: random blood sugar 204 mg dL^–1^, serum lactate 206 mmol L^–1^, blood urea nitrogen 8 mg dL^–1^ and blood pressure 135/80 mmHg. On the third day, blood sugar was not measured, lactate was 197 mmol L^–1^, blood urea nitrogen was 20 mg dL^–1^ and blood pressure was 153/87 mmHg. The skin eruption was gradually improved and the patient experienced re-epithelialization without recurrence, leaving only post-­inflammatory pigmentation after 4 weeks. A drug-induced lymphocyte stimulation test (LST) was performed 35 days after the drug insult to identify the causative agent; however, tirabrutinib was a negative result.

## Discussion

Severe drug eruptions caused by tirabrutinib are extremely rare. Including our case, there have been only three reported cases of severe drug eruptions such as Stevens–Johnson syndrome (SJS) and SJS/TEN overlap syndrome associated with tirabrutinib. The key findings from these cases are summarized in Table 1.^3,4^ In all three cases, the skin eruption initially appeared on the trunk within 2–4 weeks after starting treatment and progressed to erosions with mucosal involvement. In previous cases, skin eruption appeared approximately 2 weeks after tirabrutinib administration (on days 12 and 14). However, in the present case, it occurred 1 month later. Treatment included steroid pulse therapy, IVIg and/or plasma exchange, all of which led to improvement. While considering the patient’s advanced age, early aggressive therapy should be pursued.

**Table 1:** Literature review of cases of drug eruption caused by tirabrutinib inhibitors: summary

Reference	BTK inhibitor	Age (years)	Sex	Skin eruption type	Duration of onset	Treatment	LST
Kawano *et al*.^3^	Tirabrutinib	88	Female	SJS/TEN overlap	12 days	Steroid pulse therapy	Negative
						IVIg	
Hirano *et al*.^4^	Tirabrutinib	66	Male	TEN	14 days	1 g daily methylprednisolone	None
						Plasmapheresis	
						IVIg	
Our case	Tirabrutinib	74	Male	TEN	1 month	Steroid pulse therapy	Negative
						IVIg	

IVIg, intravenous immunoglobulin; LST, lymphocyte stimulation test; SJS/TEN, Stevens–Johnson syndrome/toxic epidermal necrolysis.

LST in two cases was negative. The negative LST results may be attributed to the effects of tirabrutinib. BTK inhibition is known to stimulate antigen-presenting activity in dendritic cells,^5^ while suppressing T-cell proliferation^6,7^ mediated by T-cell-expressed BTK. LST evaluates drug reactions based on T-cell proliferation, particularly through H3-thymidine incorporation.^8^ Therefore, the suppression of lymphocyte proliferation by tirabrutinib may lead to false-negative results in LST. Given these limitations, further research is needed to explore alternative methods for identifying causative drugs.

Additionally, BTK inhibition activates cytotoxic CD8^+^ T cells, which may contribute to the pathogenesis of SJS/TEN through rapid and widespread keratinocyte death. BTK is downstream of the B-cell receptor and is responsible for B-cell activation through the phosphorylation of downstream molecules. This B-cell activation leads to the secretion of interleukin (IL)-10, IL-35 and transforming growth factor-β, which suppress cytotoxic T-cell activity.^9^ By inhibiting BTK, tirabrutinib may induce CD8^+^ T-cell activation, resulting in keratinocyte death and contributing to the development of SJS/TEN. However, there have been no reports of severe drug eruptions such as SJS/TEN with ibrutinib, a different BTK inhibitor, and most reports involve drug eruptions with distinct features from those associated with tirabrutinib, such as neutrophilic dermatosis.^10^ This may be due to the fact that tirabrutinib is a more selective BTK inhibitor, potentially making it more prone to triggering immune responses via BTK pathways. However, further analysis is needed to draw a definitive conclusion.

## Data Availability

The data underlying this article are available in the article.
